# Preliminary Validation of the CI-FRA Checklist: A Simple Screening Tool for Measuring the Early Signs of Reading and Spelling Disorders in Italian Primary Students

**DOI:** 10.3389/fpsyg.2020.516424

**Published:** 2020-10-26

**Authors:** Sara Giovagnoli, Luigi Marotta, Sara Magri, Michela Muccinelli, Alessandra Albani, Giulia Casu, Sara Garofalo, Mariagrazia Benassi

**Affiliations:** ^1^Department of Psychology, University of Bologna, Bologna, Italy; ^2^Bambino Gesù Children Hospital (IRCCS), Rome, Italy; ^3^Azienda Unità Sanitaria Locale (AUSL) della Romagna, Emilia-Romagna, Italy

**Keywords:** reading, spelling, reading disorders, confirmatory factor analysis, checklist, early indicators

## Abstract

Although several screening tests for recognizing early signs of reading and spelling difficulties have been developed, brief and methodologically grounded tools for teachers are very limited. The present study aimed to lay the foundation for a new screening tool for teachers: the Checklist for early Indicators of risk Factors in Reading Ability (CI-FRA). The proposed checklist consists of 20 items, based on a 7-point Likert scale, and it investigates five domains: reading, writing, attention, and motor skills. Six hundred sixty-seven children were evaluated by 40 teachers during the first year of primary school and, longitudinally, in the second year. Exploratory factor analysis and confirmatory factor analysis (CFA) were applied to verify structural validity. Concurrent validity was assessed by Spearman correlation to analyze the link between CI-FRA and reading and spelling standardized tests and cognitive tests. Reliability was assessed by Cronbach α and interclass correlation coefficient. The CFA reported a three-factor structure as the optimal solution, including language (reading and writing), visuospatial attention, and fine motor skills subscales. Good reliability, good internal consistency, and acceptable test–retest indices were found. Concurrent validity was confirmed by significant correlations between CI-FRA total score and standardized reading and spelling test, as well as by correlations between CI-FRA subscales and neuropsychological standardized test scores. Preliminary evaluation of sensitivity by receiver operating characteristic curves showed that the CI-FRA score has particularly high sensitivity and specificity for word reading speed deficit. In conclusion, the results confirm that CI-FRA is a theoretically grounded and statistically valid tool that could help the teachers to screen for early signs of reading and spelling difficulties.

## Introduction

Several studies show that early intervention is crucial to correct some of the adverse effects of reading difficulties (Torgesen et al., 2001; Poskiparta et al., 2003; Elbro and Scarborough, 2004; Torgesen, 2005). For this reason, the identification of early signs characterizing children with reading and spelling difficulties is essential.

Although many standardized tests are available for clinicians to assess learning disorders, little effort has been done in literature in terms of tools dedicated to teachers. Teachers are the first adults evaluating the daily signs of progress in children, therefore having the highest chance to recognize learning disorders at an early stage. The behavioral checklists currently available (Mash and Wolfe, 2002; Wagner, 2003) are global or broad-spectrum rating scales based on parent and teachers’ ratings about the frequency and intensity of a wide range of behaviors. To our knowledge, none of these focuses on both precursors and current learning abilities as evaluated by the teacher. Furthermore, the need for new instruments dedicated to teachers has been largely demonstrated by the absence of theoretically grounded and statistically validated tools (Snowling, 2013; Catts et al., 2015). Indeed, screening tests for specific learning disorders have often proved to be inaccurate (Catts et al., 2009; Johnson et al., 2009; Compton et al., 2010). For example, longitudinal studies have shown how tests evaluating early predictors of reading difficulties resulted in high percentages of false-positive and false-negatives (Catts et al., 2015; Poulsen et al., 2017). Poulsen et al. (2017) stated that there is a methodological “dilemma”: on the one hand, preschool screening would allow early intervention, but measures to detect reading difficulties are inaccurate; on the other hand, a school-based screening test would be more accurate but would delay the intervention. In line with Poulsen et al. (2017), the present study aimed to create a fast screening tool based on different domains of evaluation and including both early indicators of learning disorders and a measure of current reading and spelling abilities.

Learning disorders are related to a complex neuropsychological profile where multiple difficulties are traced within different cognitive domains (Pennington, 2006). The multifactorial theory suggests that the etiology of learning disorders is multifactorial; i.e., it involves the interaction of multiple risks and environmental factors that impact on multiple cognitive domains (Menghini et al., 2010). According to this model, both phonological and non-phonological abilities could be impaired in subjects having reading and spelling disorders; therefore, careful early evaluation of a wide range of cognitive abilities appears to be necessary for early detection of future reading and spelling disorders.

Language-related visual abilities (such as letter knowledge, phonological awareness, and rapid automatized naming) are regarded by some authors as one of the main precursors of future reading ability (Kirby et al., 2003; Elbro and Scarborough, 2004; Landerl and Wimmer, 2008; Puolakanaho et al., 2008; Catts et al., 2009; Lervåg et al., 2009; Landerl et al., 2013; Poulsen et al., 2015), arguing that the inconsistencies among studies are related to the differences among the orthographies investigated in each study (Ziegler et al., 2010). Yet, a study by Moll et al. (2014) including a large cohort of students from five European countries ordered by grapheme-phoneme consistency (from the lowest, i.e., English, to the highest consistency, i.e., Finnish) found comparable results for different orthographies. Phonological processing and rapid automatizing naming (RAN) were both reported as important indicators for reading and spelling development. RAN was the best reading speed indicator, whereas phonological processing was the best predictor of reading accuracy and spelling. The lower the consistency of the language, the better these indices worked as predictors. Indeed, English orthography, being the less consistent one, shows a stronger predictive effect of RAN and phonological processing than all other orthographies. Italian is instead characterized by high grapheme–phoneme consistency (transparent orthography) with no irregular words, no non-homographic homophones, and no alternative acceptable phonological ways of spelling words (Zoccolotti et al., 1999). Considering phonological abilities, a recent study demonstrated that phonological awareness is a strong predictor for word reading in Italian language (Holopainen et al., 2020).

Although there is general agreement on the role of phonological abilities as an early indicator of reading and spelling disorders, the role of other predictive factors is more controversial. A group of studies pointed out that visuospatial attention could be considered a general precursor of reading difficulties both in Italian and French cohorts (Facoetti and Molteni, 2001; Valdois et al., 2004; Bosse et al., 2007; Bosse and Valdois, 2009; Facoetti et al., 2010). Indeed, visuospatial attention was found to predict irregular word reading independently from phoneme awareness. However, recent studies found a double dissociation between dyslexia and visuospatial attention, thus opposing the importance of attention in predicting reading disorders (Lukov et al., 2015).

Further controversy is related to the predictive role of motor skills for children’s school readiness (e.g., Grissmer et al., 2010), showing a link with intellectual skills. In particular, Grissmer et al. (2010) showed that both attention and fine motor skills measured at kindergarten are important developmental predictors of later academic achievement. Cameron et al. (2016) also emphasized how motor skills are implicated in children’s self-regulation and their future reading, spelling, and numeracy. In a study by Roebers and Jäger (2014), fine motor skills were found to have a noticeable predictive power for school achievement and literacy, but strictly associated with executive functions. Other studies (e.g., Viholainen et al., 2002) confirmed that motor and language problems are often interconnected. Moreover, fine motor skills have been found to be associated to executive functions (Roebers and Jäger, 2014), and some studies demonstrated that they are predictors of written expression achievement (Carlson et al., 2013). Besides, some studies demonstrated that, in preschool and early elementary classrooms, motor coordination, executive functions, and visuospatial processes are combined with other skills to form the basis for children’s successful learning (Pagani and Messier, 2012; Cameron et al., 2016).

Taken together, this evidence suggests that early evaluation of language, visuospatial attention, and fine motor skills should be considered for the early identification of children at risk of learning disorders (Gabrieli and Norton, 2012; Cameron et al., 2016).

Moving from this evidence and the multifactorial theory (Menghini et al., 2010), we aimed to lay the foundation for a new screening tool for teachers: the Checklist for early Indicators of risk Factors in Reading Ability (CI-FRA). This checklist is based on the evaluation of early precursors and current state of reading and spelling difficulties through the analysis of five domains (language, reading, spelling, attention, and motor skills).

Reading and spelling difficulties are knowingly overlapping in learning disorders. At least three plausible theoretical models can explain the heterogeneity of early signs of reading and spelling difficulties along a continuum of specificity. In line with the multifactorial theory, a first model includes altogether the learning difficulties evaluated as explained by a general factor expressing the severity of the learning disorder (minimum specificity); a second model distinguishes the specificity of each specific domain investigated with factors explaining the variability of each domain (maximum specificity); and a third model accomplishes the possible overlapping as well as the specificity of the domains investigated and includes a number of factors that are inferior to the number of domains, i.e., macrodimension (intermediate specificity).

Because no previous study validated such a theoretical model, a confirmatory factor analysis (CFA) was used to test the dimensionality of early signs of reading and spelling difficulties. Moreover, the validity, sensitivity, and reliability of the CI-FRA were statistically tested.

## Materials and Methods

### Participants and Procedure

Participants were recruited through contact and direct agreement with the school managers of the 23 primary schools located in the province of Forlì-Cesena, in the Emilia Romagna region (North of Italy). Italian education is based on a state school system and follows the same rules and the same educational curriculum for all the regions. The primary school is the first compulsory school, and it is commonly preceded by 3 years of kindergarten. The primary school lasts 5 years, and the first year starts at 6 years. During the first 3 months of the first year, the pupils learn reading and spelling, and at the end of grade 2, they are expected to be proficient. All the data were collected between the end of February 2017 and June 2017 (after 5–6 months from the beginning of the academic year). A total of 667 children (310 females) attending grade 1 of the primary school [mean age is 6.64 years, standard deviation (SD) = 0.28 years] participated in the study.

Institutional review boards approved the study, and both parents gave written informed consent. In the case of single parent, we asked the responsible parent for the informed consent.

The exclusion criteria adopted were those recommended by the Consensus Conference on Specific Learning Disorders promoted by the Italian National Institute of Health (Lorusso et al., 2014) for diagnosis of developmental dyslexia. Participants with an IQ lower than 70 and having referred sensory disability were excluded from the study. In order to evaluate the predictive, concurrent validity, and sensitivity of CI-FRA, we selected from the total sample a subsample of 106 children (males = 64; mean age = 6.6 years, SD = 0.28 years) recruited from two schools who agreed to participate in the follow-up evaluation. This subsample was assessed in regard to specific cognitive measures (general cognitive functioning, visual attention, phonological skills) simultaneously with CI-FRA administration during the first grade and at the end of the second grade (i.e., September 2018) reevaluated for reading and spelling abilities with standardized tests. A group of expert psychologists was responsible for the assessment and the relationships with the teachers and parents. The standardized cognitive tests were chosen because they are considered the main indicators for Italian reading and spelling acquisition. These indications have been published in official public documents promoted by the Italian National Institute of Health as the Consensus Conference on Specific Learning Disorders for diagnosis of developmental dyslexia (Istituto Superiore di Sanità, 2011). Within the subsample, 86 children (males = 51; mean age = 6.6 years, SD = 0.29 years) had a second evaluation by their teachers at CI-FRA (first at the end of February 2017 and a second time at the end of May 2017) to measure test–retest reliability.

No significant differences were found between the total sample and the two subsamples for demographics characteristics or gender. As Table 1 shows, no differences were found for maternal education level or for paternal ones between the three samples. The percentages of mothers and fathers who had school difficulties in the past were not significantly different between the three subsamples, as well as no differences were found for gender distribution (Table 1).

**TABLE 1:** Demographic characteristics of the three samples used in the study.

			**Entire sample**	**Cognitive measures sample**	**Test–retest sample**	**χ^2^**
Maternal	Education (%)	Primary and secondary school	102 (25.0%)	14 (23.7%)	14 (23.7%)	0.079
Variables		High school and university	306 (75.0%)	45 (76.3%)	45 (76.3%)	
	School difficulties (%)	Yes	19 (4.7%)	1 (1.7%)	2 (3.4%)	1.23
		No	387 (95.3%)	58 (98.3%)	56 (96.6%)	
Paternal	Education (%)	Primary and secondary school	132 (33.1%)	15 (25.4%)	16 (27.6%)	1.88
Variables		High school and university	267 (66.9%)	44 (74.6%)	42 (72.4%)	
	School difficulties (%)	Yes	19 (4.7%)	4 (6.9%)	4 (6.9%)	0.85
		No	382 (95.3%)	54 (93.1%)	54 (93.1%)	
Children	Gender	Males	357 (53.5%)	64 (60.4%)	51 (59.3%)	2.47
variables		females	310 (46.5%)	42 (39.6)%	35 (40.7%)	

According to the Italian school general population, it is common to have a high percentage of bilingual pupils; therefore, we decided to include in the whole sample also the bilinguals and ask the teachers to have additional information about their exposure to the Italian language. In the total sample, 81.5% of the pupils were monolingual and used Italian as their language, whereas 18.5% of the total sample were bilingual. Within the bilinguals, 46.4% had the Italian language as L1, and 54.6% as L2. The languages more common after Italian were Arabic and Albanian (16.7 and 13.8%, respectively), whereas the most common languages as L2 after Italian were Arabic, Albanian, and Romanian (7.4, 9.5, and 9.3%). The 8.7% of bilinguals had been in Italy for over 3 years, and the 4.8% (only five children) had been in Italy for less than 3 years; all the others were born in Italy. Teachers reported that 87.8% of the total sample had a good oral comprehension ability, 11.7% showed a sufficient ability, whereas the 0.5% had difficulties (all those children are bilinguals). Most of the parents’ participants (71%) had finished high school, college, or university, and 95.3% declared that they did not have difficulty at school. Finally, 87.3% stated that no one in the family presented specific learning disorders.

### Instruments

#### CI-FRA Checklist

The CI-FRA checklist was created by a team of psychologists and speech therapists who worked in two different research teams and jointly collaborated to the project.

Three steps were settled for the development of the final version of the checklist. In a first step, three psychologists and one speech therapist, two are coauthors in the present articles, prepared a list of possible items on the basis of multifactorial model of dyslexia (Pennington, 2006; Menghini et al., 2010; Ziegler et al., 2019). According to the multifactorial model of dyslexia, the phonological memory and phonological awareness, as well as visual attention functions and motor skills, were considered important predictive factors of reading and spelling difficulties. Therefore, for each one of these cognitive domains, we included a specific set of items. In a second step, three additional psychologists and one speech therapist revised the set of items by selecting the most relevant items for each one of the cognitive domains by ordering them for their importance. In the last step, a group of 60 teachers evaluated the adequacy of the checklist by excluding some items because of their redundancy and by ameliorating the terms used in some others items (the specialistic language used in some cases was not sufficiently clear for the teachers).

The final version of the CI-FRA checklist comprises 20 items that measure the student’s learning disorders, as referred by the teacher’s teaching experience (Table 2). The teacher (or the team of teachers) is asked to evaluate each student’s difficulties in the different domains by comparing him/her to an ideal reference “average student” based on his/her teaching experience. The frequency of occurrence of each problematic behavior is measured using a seven-point Likert scale ranging from 1 (never observed) to 7 (often observed). The checklist has to be compiled by the teacher; as a first administration point, it is recommended to administer the CI-FRA 3 to 4 months after the beginning of the Academic year, then it can be used every 5 months to monitor the developmental changes.

**TABLE 2:** CI-FRA checklist.

	**Italian version**	**English version**
Item 1	L’alunno fatica ad esprimersi oralmente (le difficoltà possono riguardare gli aspetti fonologici, articolatori e/o la produzione morfosintattica)	The student has difficulty in oral expression (difficulties can concern phonological and articulatory aspects and/or morphosyntactic production).
Item 2	L’alunno per esprimersi utilizza poche parole e sempre le stesse (ampiezza del vocabolario limitata)	In oral communication, the student uses a limited number of words and tends to use always the same words (restricted vocabulary).
Item 3	L’alunno fatica a costruire una parola dai singoli fonemi (sintesi fonemica) o a individuare i fonemi che compongono la parola (segmentazione fonemica)	The student struggles to create a word starting from separated phonemes (phonemic synthesis) or to identify the phonemes that compose the word (phonemic segmentation).
Item 4	L’alunno legge più lentamente rispetto ai coetanei	The student has a lower reading speed compared to peers.
Item 5	L’alunno, quando legge, commette molti errori	When reading, the student makes many mistakes.
Item 6	L’alunno, quando legge, commette errori di confusione tra lettere che hanno un suono simile (es. p-b, c-g, f-v) o che sono visivamente simili (es. m-n, b-d, a-e)	When reading, the student makes confusion errors between letters that have a similar sound (e.g., p-b, c-g, f-v) or that are visually similar (e.g., m-n, b-d, a-e).
Item 7	L’alunno mostra difficoltà nella lettura delle parole bisillabiche piane	The student struggles in reading simple disyllabic words.
Item 8	L’alunno, quando legge, si affatica facilmente	When reading, the student gets tired quickly.
Item 9	La grafia dell’alunno risulta poco leggibile	The student’s handwriting is difficult to read.
Item 10	L’alunno impugna la matita/penna con difficoltà o in modo inadeguato	The student holds the pencil/pen having difficulty or inadequately.
Item 11	L’alunno mostra difficoltà nella gestione del foglio (rispetto delle righe, dei quadretti, i margini)	The student shows difficulties in managing spaces in the paper (poor awareness of lines, squares, margins of the paper).
Item 12	L’alunno mostra difficoltà nella motricità fine (es. usare le forbici, allacciare bottoni)	The student shows difficulties in fine motor skills (e.g., using scissors, fastening buttons).
Item 13	L’alunno, quando scrive, commette errori di confusione tra lettere che hanno un suono simile (es. p-b, c-g, f-v) o che sono visivamente simili (es. m-n, b-d, a-e)	When writing, the student confuses letters that have a similar sound (e.g., p-b, c-g, f-v) or that are visually similar (e.g., m-n, b-d, a-e).
Item 14	L’alunno, quando scrive, tende ad invertire le lettere, ad esempio gli capita di scrivere “la” invece che “al”	When writing, the student tends to reverse the letters (e.g., he writes “fo” instead of “of”).
Item 15	L’alunno mostra difficoltà nella scrittura delle parole bisillabiche piane	The student has difficulties in writing simple disyllabic words.
Item 16	L’alunno si distrae facilmente	The student is easily distracted.
Item 17	L’alunno si affatica facilmente	The student gets tired easily.
Item 18	L’alunno si muove molto sulla sedia mentre deve eseguire i compiti, giocherella con gli oggetti presenti sul tavolo, ecc…	The student moves a lot on the chair while doing homework, plays with the objects on the table, etc.
Item 19	L’alunno impiega molto più tempo degli altri per portare a termine le attività in classe	The student takes much longer than others to complete classroom activities.
Item 20	L’alunno fatica nei compiti che riguardano la memoria (esempio poesie, mesi, filastrocche)	The student has difficulties in memory tasks (poems, nursery rhymes, months).

The checklist has been developed to measure five dimensions or subscales (phonological awareness and verbal memory, reading, spelling, motor skills, attention): language and verbal memory subscale includes items 1 to 3 and item 20; reading subscale includes the items 4 to 8, fine motor skills is composed of items 9 to 12, spelling dimension includes items 13 to 15, and the last dimension regards attention abilities and is composed of items 16 to 19. The scores of the subscales are obtained as the mean of the items included in each subscale. The total score is obtained as the sum of all the items.

Moreover, the CI-FRA is accompanied with an interview in which the teacher is asked to specify the presence of bilingualism and the familiarity for learning disorders (e.g., the parental education levels and possible relatives with learning disorders).

The CI-FRA is not only available as a paper-and-pencil tool but is also in digital format that includes the formula to calculate the scores for each student and to show the changes in each area graphically. Moreover, the teacher could have a graphical representation of the entire class.

#### Standardized Cognitive Tests

##### Raven’s colored progressive matrices (CPM)

The test evaluates the non-verbal intellectual abilities, such as logical ability, visuospatial components, and the ability to analyze abstract images according to similarity, dissimilarity, numerical progression, and size (Raven, 1994). The test consists of 36 items, and the subject is required to look at an incomplete figure and identify the missing piece between 4 and 8 alternatives. The subject total performance represents the subject total score.

##### Digit span (Wechsler intelligence scale for children-IV subtest)

This subtest measures short-term auditory memory and working memory’s ability (Wechsler, 2005). The subject’s task is to listen and repeat a sequence of numbers. The sequence increases in length at each trial. Forward and backward digit span abilities are tested. In the backward task, the participant has to recall the sequence in reverse order (working memory). Subject’s total score is obtained by summing forward and backward correct responses.

##### RAN colors

The task measures automatization naming ability that is a competence related to language abilities (De Luca et al., 2005). “Colors” condition is composed of a sequence of colored dots, and the subject’s task is to name the colors as fast as possible. Total time and total correct answers represent the subject’s scores in speed and accuracy.

##### Visual search (VS) objects

The task is used to evaluate visual attention ability (De Luca et al., 2005). “Objects” condition is composed of matrices of different figures (stars, pears, cows, trains, and hands) painted in a paper, and the subject’s task is to identify and mark with a pen the target figure (star) as quickly as possible. Total time and total correct answers represent the subject’s score in speed and accuracy.

##### Metaphonological skills (CMF)

This test allows for evaluating the development of metaphonological skills in children from 5 to 11 years (Marotta et al., 2008). Metaphonological abilities represent important prerequisites for adequate learning and development of reading and spelling abilities and are related to language competences. In this study, to evaluate the different types and levels of phonological awareness, we used the following subtests: *segmentation test*, in which it is required to say, in the correct sequence, the segmental units (syllables), which constitute the different words (segmentation); *phonemic synthesis test*, in which the word resulting from the fusion of a series of phonemes pronounced by the examiner in the correct sequence (synthesis); *deletion of the initial syllable test*, in which it is required to pronounce a word without the initial syllable (manipulation); FAS test (verbal fluency test with phonemic facilitation), in which it is required to say as many words as possible starting with the same letter/sound (classification). The sum of correct answers in each subtest represents the subject’s scores.

##### Non-word repetition (NWR)

This task involves different processes, including phonological memory and speech production and evaluates the ability to listen and repeat unusual sound patterns (non-word) (Subtest of PROMEA test; Vicari, 2007). This test consists of 40 non-words, and non-words can have high or low resemblance according to the number of changed letters compared to an existing Italian word. The sum of correct answers represents the subject’s score.

#### Reading and Spelling Standardized Tests

Standardized batteries for Italian reading and spelling ability (DDE-2, Sartori et al., 2007; MT, Cornoldi et al., 2018) were used to test the presence of specific reading disability and specific spelling disability. Within the battery, accuracy and speed in reading were evaluated by using a written text (MT, Cornoldi et al., 2018). Spelling abilities were tested as the accuracy in a test (DDE-2, Sartori et al., 2007) that requires the child to write non-words list. Fine motor skills were tested by a test for evaluating the speed and fluidity of handwriting in which the child is asked to produce specific graphemes (lelele) as much as he can and as quickly as possible (BVSCO, Tressoldi et al., 2013). Raw scores were transformed into *z* standardized scores according to normative data. For each test, the children having a score equal to or less than 1.5 SDs are considered as having a deficit in the specific learning domain (reading, spelling, or fine motor skills).

### Statistical Analysis

To assess the CI-FRA structural validity, exploratory factor analyses (EFA) and CFA were conducted on two randomly created subsamples. The sample size was established *a priori* as to have a subject to an item ratio of 10:1 in the EFA (Nunnally, 1978) and at least 10 observations for each freely estimated model parameter in the CFA (Kline, 1998).

Exploratory factor analyses with principal axis factoring (PAF) and Promax rotation was performed on the first subsample (*n* = 200). PAF is an extraction method generally used when testing a theoretical model method (Tabachnick and Fidell, 2001) as in this study where we expected a model-structure fitting the multifactorial model theory (Pennington, 2006; Menghini et al., 2010). Kaiser–Meyer–Olkin (KMO) measure of sampling adequacy test and Bartlett test of sphericity were used to check whether the data were adequate to apply factor analysis. Factors were extracted based on Kaiser’s criterion Kaiser (1960) of eigenvalue higher than 1. Items with loadings greater than 0.40 and cross-loadings less than 0.10 were considered for inclusion in a factor.

Confirmatory factor analysis was performed on the second subsample (*n* = 467) to test the factor model that resulted from EFA. Model parameters were estimated using the robust maximum likelihood method. The closeness of the hypothesized model to the empirical data was evaluated through the following goodness-of-fit indices: χ^2^, Satorra–Bentler scaled χ^2^ statistic (S-B χ^2^); root mean square error of approximation [RMSEA, cutoff < 0.10, upper bound of the 90% confidence interval (CI) ≤ 0.10]; standardized root mean square residual (cutoff < 0.10); and comparative fit index (CFI, cutoff > 0.90) (Weston and Gore, 2006).

Aiming to evaluate possible alternative models explaining specificity or overlapping between the investigated domains, the three-factor model obtained by EFA and confirmed by CFA was compared to a one-factor model solution and to a five-factor model solution by CFA.

The predictive validity and the concurrent validity were verified by correlation analysis between the CI-FRA and standardized measures of reading and spelling abilities, and between CI-FRA and standardized measures general cognitive functioning and phonological skills that served as prerequisites for reading–spelling in a subsample of 106 participants. Expecting correlations with a moderate effect size, this sample size was considered adequate to have approximately 95% power (α = 0.05, two-tailed) to reject the null hypothesis.

On the same subsample, test sensitivity was evaluated by receiver operating characteristic (ROC) curves applied for each learning disorder (reading and spelling), using as state variable the qualitative results of the standardized test for reading and spelling. For each learning disorder, different standardized clinical tests were used. Children scoring equal to or less than the clinical cutoff score at the specific reading and spelling tests (1 SD for reading text, 2 SD for word lists) were registered as “below the norms” score, and these subjects were considered in the “clinical group.” The category clinical group was used as a reference category in the state variable, whereas the CI-FRA subscale scores were the test variables.

Internal consistency reliability was assessed by calculating Cronbach α (cutoff ≥ 0.70; Nunnally, 1978) and corrected item-total correlations (cutoff ≥ 0.30; Streiner and Norman, 2008). Test–retest reliability over a 3-month period was assessed in a subsample of 86 participants by calculating the intraclass correlation coefficient (ICC) with a two-way random-effects (absolute agreement) model (cutoff ≥ 0.70; Streiner and Norman, 2008). This sample size was established *a priori* to detect an expected large effect size with a power of 0.85 or greater and α = 0.05 (two-tailed).

Interpretation of results was based on both statistical significance (significant level set at *p* < 0.05) and measures of effect size, with Spearman ρ of 0.10 considered small, 0.30 medium, and 0.50 large, and Cohen *d* of 0.20 considered small, 0.50 medium, and 0.80 large (Cohen, 1988). Sample sizes were calculated *a priori* with the statistical software G^∗^Power 3 (Faul et al., 2007). CFA was performed using LISREL 8.80 (Scientific Software International, Lincolnwood, IL, United States); all other analyses were performed with IBM SPSS 25 (SPSS Inc., Chicago, IL, United States).

## Results

### Structural Validity

The EFA run on the first sample (*n* = 200) yielded three factors explaining 74.41% of the variance (Table 3). The KMO measure of sampling adequacy proved to be extremely good (KMO = 0.92; Hutcheson and Sofroniou, 1999), and Bartlett test of sphericity proved to be highly significant (*p* < 0.001). According to Kaiser criterion Kaiser (1960), three factors had an eigenvalue >1 and explained 74.4% of the total variance. All the items respect the inclusion criteria in a factor (item’s loadings greater than 0.40 and a cross-loading less than 0.10). Table 3 shows the items’ loading for the 3-factor solution.

**TABLE 3:** EFA factor loadings (*n* = 200), and CFA goodness-of-fit indices (*n* = 467).

**Item content**	**Mean ± SD**	**F1**	**F2**	**F3**
7. The student struggles in reading simple disyllabic words.	1.21 ± 1.62	**0.98**	−0.02	−0.09
5. When reading, the student makes many mistakes.	1.55 ± 1.77	**0.98**	0.03	−0.07
6. When reading, the student makes confusion errors between letters that have a similar sound (e.g., p-b, c-g, f-v) or that are visually similar (e.g., m-n, b-d, a-e).	1.46 ± 1.70	**0.97**	0.03	−0.06
15. The student has difficulties in writing simple disyllabic words.	1.29 ± 1.70	**0.96**	−0.04	−0.06
4. The student has a lower reading speed compared to peers.	1.81 ± 1.90	**0.90**	0.13	−0.08
3. The student struggles to create a word starting from separated phonemes (phonemic synthesis) or to identify the phonemes that compose the word (phonemic segmentation).	1.35 ± 1.75	**0.87**	−0.18	0.20
13. When writing, the student confuses letters that have a similar sound (e.g., p-b, c-g, f-v) or that are visually similar (e.g., m-n, b-d, a-e).	1.68 ± 1.71	**0.86**	0.00	0.08
20. The student has difficulties in memory tasks (poems, nursery rhymes, months).	1.37 ± 1.70	**0.74**	0.14	0.00
1. The student has difficulty in oral expression (difficulties can concern phonological and articulatory aspects and/or morphosyntactic production)	1.42 ± 1.85	**0.65**	−0.21	0.39
8. When reading, the student gets tired quickly.	1.36 ± 1.77	**0.63**	0.34	−0.03
2. In oral communication, the student uses a limited number of words and tends to use always the same words (restricted vocabulary).	1.54 ± 1.89	**0.62**	−0.13	0.32
14. When writing, the student tends to reverse the letters (e.g., he writes “fo” instead of “of”).	1.27 ± 1.58	**0.59**	0.08	0.11
16. The student is easily distracted.	2.43 ± 1.93	−0.08	**0.88**	0.12
17. The student gets tired easily.	1.78 ± 1.86	0.17	**0.81**	−0.02
18. The student moves a lot on the chair while doing homework, plays with the objects on the table, etc.	2.18 ± 1.94	−0.24	**0.80**	0.24
19. The student takes much longer than others to complete classroom activities.	2.05 ± 2.12	0.33	**0.67**	−0.10
11. The student shows difficulties in managing spaces in the paper (poor awareness of lines, squares, margins of the paper).	1.39 ± 1.71	0.05	0.12	**0.79**
12. The student shows difficulties in fine motor skills (e.g., using scissors, fastening buttons).	1.32 ± 1.68	0.01	0.17	**0.79**
9. The student’s handwriting is difficult to read.	1.02 ± 1.42	0.04	0.15	**0.71**
10. The student holds the pencil/pen having difficulty or inadequately.	0.92 ± 1.39	0.04	0.01	**0.57**
**Eigenvalues**		11.98	2.02	0.88
**Eigenvalues after Promax rotation**		11.11	7.3	7.6
**Cronbach α**		0.97	0.91	0.89

**Fit indices**	**χ^2^ (167)**	**S-B χ^2^ (167)**	**RMSEA [90% CI]**	**CFI**

	1,534.07*	881.143*	0.096 [0.090, 0.10]	0.98

*p < 0.001. In bold the highest items’ loading for the 3-factor solution. S-B X^2^, Satorra-Bentler scaled X^2^; RMSEA, Root Mean Square Error of Approximation; CFI, Comparative Fit Index.

The first factor extracted is composed of 12 items and includes the items representing behaviors related to phonological abilities (1, 2, and 3), reading (4, 5, 6, 7, and 8), and spelling ability difficulties (13, 14, and 15). In addition, item 20 (created to be representative of behaviors related to verbal memory difficulties) was included in Factor 1. The second factor included the four items describing behaviors typically related to attention difficulties (16, 17, 18, and 19). Finally, the third factor included four items (9, 10, 11, and 12) representing potential fine motor skills difficulties. Skewness and kurtosis computed on the three-factor scores indicated approximately normal univariate distributions, being lower than |2| (Tabachnick and Fidell, 2006); skewness (SE = 0.17) was between 0.58 and 1.27, and kurtosis (SE = 0.34) between −0.11 and 0.95.

**FIGURE 1:**
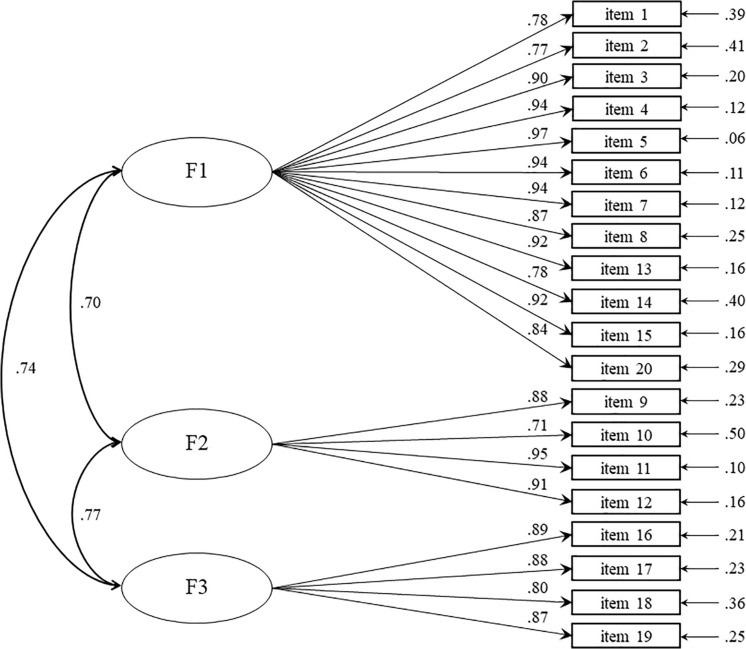
Measurement model with standardized parameters (*n* = 467).

This three-factor model was tested on the second sample (*n* = 467) using CFA. Results indicated an acceptable fit to the data, with all indices close to the expected value (Table 3). Each item loaded highly (>0.70) and significantly (*p* < 0.001) on its designated factor, with factor loadings in the 0.71–0.97 range and error variances in the 0.06–0.50 range (Figure 1). Latent variables were positively, strongly correlated (*p* < 0.001).

In order to evaluate possible alternative models explaining specificity or overlapping between the investigated domains, the three-factor model confirmed by CFA was compared to the one-factor model solution (representing unique general factor as an expression of the severity of the difficulty) and to a five-factor model solution (representing the specificity of each specific domain investigated) by CFA (Table 4).

**TABLE 4:** CFA goodness-of-fit indices (*n* = 467) for the one-factor model, the three-factor model, and the five-factor model.

**Model**	**n. par**	**χ^2^ (*df*)**	**CFI**	**RMSEA (CI)**	**ECVI (CI)**	**AIC**	**CAIC**	**BIC**
One-factor	40	3,203.63* (170)	0.752	0.196 (0.190, 0.202)	7.046 (6.660 7.449)	3,283.63	3,489.48	3,449.48
Three-factor	63	1,534.07* (167)	0.981	0.096 (0.090, 0.10)	2.161 (1.927; 2.326)	1,007.14	1,331.36	1,268.36
Five-factor	70	1,444.53* (160)	0.981	0.100 (0.093, 0.105)	2.228 (1.990; 2.395)	1,038.07	1,398.31	1,328.31

N. par, number of parameters estimated; CFI, Comparative Fit Index; RMSEA, Root Mean Square Error of Approximation; ECVI, Expected Cross-Validation Index; AIC, Akaike Information Criterion; CAIC, consistent Akaike Information Criterion; BIC, Bayesian Information Criterion. *p < 0.05.

All the models have shown significant χ^2^ indices, suggesting that no good model fits. However, as suggested by many authors, the χ^2^ test is widely recognized to be problematic (Jöreskog, 1969; Kim and Mueller, 1978; Bentler, 1990). It is sensitive to sample size, and it becomes more difficult to obtain a non-significant test as the number of cases increases. Analyzing the difference between the CFI in the three models, it is possible to see as the three- and five-factor models have very similar CFIs, and in both cases, CFI that resulted above the cutoff generally indicated a sign of good model fit (>0.95; Hu and Bentler, 1999). The CFI of the one-factor model is lower than the threshold of 0.95 and is significantly lower than those found for the three- and five-factor models. RMSEA indices resulted above the threshold of 0.06 commonly used to indicate a good model fit (Hu and Bentler, 1999) in all three models. However, according to the criteria suggested by MacCallum et al. (1996), a RMSEA of less than 0.10 can be considered as an indicator of a mediocre fit of the model as in the case of the three- and five-factor models, whereas, also using this criterion, the RMSEA for the one-factor model suggested a non-adequate fit model (with a RMSEA equal to 0.19). As regards Expected Cross-Validation Index (ECVI), no specific parameters for model acceptance or rejection exist for ECVI values; instead, this statistic assesses the likelihood that a model cross-validates across similar sized samples from the same population. In other words, the ECVI is used to compare competing models, with smaller values suggestive of greater generalizability (Byrne, 1998). When a model has a lower ECVI value, and when the ECVI value for a competing model is above the upper 90% confidence limit of the first model, it can be concluded with greater confidence that the first is the better of the two competing models (O’Rourke and Hatcher, 2013). In this case, the ECVI of the one-factor model was above both of the upper 90% CI of the three-factor model and the upper 90% CI of five-factor model; these results suggested preferring the three- and five-factor models to the one-factor one. As regards the parsimony fit indices, it is possible to note as the three-factor model showed the lowest values for Akaike Information Criterion, Bayesian Information Criterion, and consistent Akaike Information Criterion, whereas the five-factor solution showed the highest but similar values, the one-factor model presented significantly highest values for all the indices. These results suggested preferring the three-factor model. Observing the number of estimated parameters, the most parsimonious model was the one-factor model, with 40 estimated parameters, followed by the three-factor model (63) and by the five-factor model (70).

Summarizing all the information on the model fit indices, both for the three-factor model and for the five-factor model, the fit to the data was acceptable; in fact, both the three- and five-factor models showed fit indices close to the expected value. The one-factor model showed the worst fit indices and resulted to be the model that less fit the data. Despite that the three- and five-factor models resulted to be very alike, obtaining very similar fit indices, the evaluation of the information criteria indices suggested to prefer the three-factor model to the five-factor model.

Finally, following the parsimony principle (essential because it helps discriminate the signal from the noise, allowing better prediction and generalization to new data; Vandekerckhove et al., 2015) and bearing in mind that the one-factor model has shown the worst fit indices with respect to the three- and five-factor models, the three-factor model, showing the best fit indices and having 63 parameters estimated (against the 70 of the five-factor model), turned out to be the model that fits the data better and that better explains the dimensionality of the analyzed data.

However, because latent variables in the three-factor model were highly correlated, a higher-order model with one second-order general factor and three first-order factors was also estimated. Such a second-order model was statistically equivalent to the model with three correlated factors, thus yielding exactly the same number of estimated parameters, fitted residuals and model fit statistics. The second-order factor loadings associated with the general factor were 0.82 for F1, 0.85 for F2, and 0.90 for F3 (*p* < 0.001). The unique first-order factor residual variances were all positive and significant, being 0.33 for F1, 0.27 for F2, and 0.19 for F3 (*p* < 0.001). Because the amount of variance associated with a first-order factor’s residual decreases as the first-order factor loading onto the general factor increases, a statistically significant residual variance indicates that a dimension is, at least partly, unique or separable (Gignac and Kretzschmar, 2017). Altogether, results support the plausibility of both a general CI-FRA factor and three unique first-order factors, corresponding to phonological, reading and spelling abilities, attention competences, and fine motor skills. The three first-order factor scores provide information about teacher-assessed abilities in specific domains, whereas the global (second-order) CI-FRA score provides summary information on students’ learning disorders as referred by the teacher.

### Reliability

Reliability estimates were adequate. In the first (*n* = 220) and second (*n* = 467) subsamples, Cronbach α’s were 0.97 and 0.98 for Factor 1, 0.89 and 0.92 for Factor 2, and 0.91 and 0.92 for Factor 3, respectively. All corrected item-total correlations were >0.50, being in the 0.74–0.94 range for Factor 1, 0.57–0.87 for Factor 2, and 0.74–0.89 for Factor 3. Test–retest reliability estimate over a 3-month period (*n* = 68) was acceptable for all the three factors. ICC of 0.73 (95% CI [0.66, 0.79]) was found for Factor 1, ICC of 0.69 (95% CI [0.61, 0.76]) for Factor 2, and an ICC of 0.67 (95% CI [0.59, 0.74]) for Factor 3.

### Predictive and Concurrent Validity and Sensitivity

Spearman ρ correlation analysis evidenced significant positive correlations between CI-FRA and accuracy and speed in standardized reading test and between CI-FRA scores and accuracy in spelling (Table 5).

**TABLE 5:** Spearman ρ correlation analysis results to evaluate concurrent validity between CI-FRA subscales and total score and the different cognitive measures.

		**CI-FRA**					
		**Language**	**Reading**	**Spelling**	**Motor skills**	**Visuospatial attention**	**Total score**
**Cognitive measures**	CMF_synthesis	0.500**	0.617**	0.551**	0.388**	0.550**	0.590**
	CMF_deletion	0.491**	0.567**	0.473**	0.369**	0.492**	0.534**
	CMF_segmentation	0.461**	0.540**	0.497**	0.402**	0.455**	0.534**
	CMF_FAS	0.609**	0.701**	0.550**	0.550**	0.637**	0.671**
	NWR	0.470**	0.535**	0.493**	0.254**	0.446**	0.496**
	Digit Span	0.377**	0.449**	0.460**	0.257**	0.482**	0.475**
	RAN_speed	0.173	0.289**	0.287**	0.208*	0.270**	0.293**
	RAN_accuracy	0.052	0.010	0.024	0.030	0.066	0.041
	VS_speed	0.137	0.084	0.085	0.027	0.039	0.095
	VS_accuracy	0.090	0.119	0.089	0.162	0.111	0.116
	CPM	0.443**	0.461**	0.429**	0.360**	0.371**	0.469**
	Text reading_speed	0.333**	0.511**	0.437**	0.324**	0.388**	0.444**
	Text reading_accuracy	0.494**	0.580**	0.573**	0.474**	0.520**	0.574**
	Words reading_speed	0.336**	0.544**	0.447**	0.363**	0.442**	0.478**
	Words reading_accuracy	0.487**	0.562**	0.561**	0.345**	0.574**	0.570**
	Spelling_accuracy	0.411**	0.467**	0.444**	0.263**	0.434**	0.460**
	Handwriting_fluidity	0.280*	0.416**	0.264*	0.332**	0.399**	0.383**

CMF_synthesis, Metaphonological skills_Phonemic Synthesis; CMF_deletion, Metaphonological skills_Deletion of the initial syllable; CMF_segmentation, Metaphonological skills_Segmentation test; CMF_FAS, Metaphonological skills_Verbal fluency test with phonemic facilitation; Digit Span, Digit Span, WISC-IV subtest; VS_speed, Visual Search Objects_speed; VS_accuracy, Visual Search Objects_accuracy; RAN_speed, Rapid Automatization Naming Colors_speed; RAN_accuracy, Rapid Automatization Naming Colors_accuracy; NWR, Non-Word Repetition; CPM, Raven’s Colored Progressive Matrices; Text reading_speed, speed in reading a written text (MT, Cornoldi et al., 2018); Text reading_accuracy, accuracy in reading a written text (MT, Cornoldi et al., 2018); Words reading_speed, speed in reading a words list (DDE-2, Sartori et al., 2007); Words reading_accuracy, accuracy in reading a words list (DDE-2, Sartori et al., 2007); Spelling_accuracy, accuracy in writing non-words list (DDE-2, Sartori et al., 2007); Handwriting_fluidity, fluidity in handwrite productions (BVSCO, Tressoldi et al., 2013). **p < 0.01, *p < 0.05.

Spearman ρ correlation analysis between CI-FRA subscales and total score and the different cognitive measures showed significant correlation with phonological awareness scales (CMF), NWR, digit span, naming colors (RAN), VS, and Raven scale (CPM). As Table 5 shows, all the CI-FRA subscales and the total CI-FRA score were correlated with the phonological awareness scale (CMF) subtests [phonemic synthesis, deletion, segmentation and verbal fluency test with phonemic facilitation (FAS)]. Significant positive correlations were found also between CI-FRA and Non-Word Repetition, as well as for CI-FRA and Digit Span test. Speed in naming colors (RAN) was significantly correlated with all the CI-FRA subtests and the CI-FRA total score except for language subscale, whereas the accuracy of RAN was not correlated with CI-FRA subscales and total score as well as visual search (VS) speed and accuracy. Raven score (CPM) was correlated with all the CI-FRA subscales and total score. Overall, the significant correlation index varied from 0.21 to 0.70, indicating small to moderate correlations between CI-FRA and the aforementioned cognitive measures.

**FIGURE 2:**
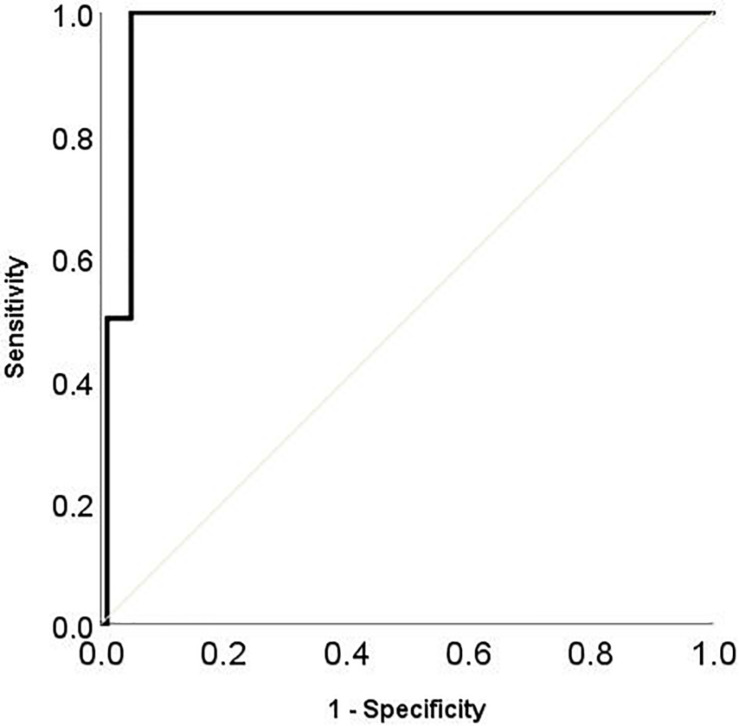
ROC curves representing sensitivity and specificity of CI-FRA total score in predicting the speed in standardized word list reading test.

The sensitivity and specificity of CI-FRA were analyzed by means of ROC curves. In the ROC curve, the state variable was created on the basis of standardized clinical measures for each specific learning disorder: those children scoring equal to or less than the clinical cutoff score at the specific reading and spelling tests (1 SD for reading text, 2 SD for word lists) were considered as having learning disorders. This clinical assessment was collected by clinicians at the end of second grade. Considering the reading abilities; we used two gold standard tests for the reading and spelling disorder diagnosis: word list test and text reading test (including both accuracy and speed). The standardized tests evidenced that the 4% of children (4/102) had deficit in word reading speed, and the 5% (5/100) had spelling deficit in non-word spelling test. In details, the ROC curves’ areas were all significant (Word List Reading Speed Area under the ROC curves = 0.97; *p* = 0.001; word list reading accuracy area under the ROC curves = 0.89; *p* = 0.001; text reading speed area under the ROC curves = 0.77; *p* = 0.001; text reading accuracy area under the ROC curves = 0.88; *p* < 0.001; spelling area under the ROC curves = 0.80; *p* = 0.020), indicating a promising applicability of CI-FRA as screening test for reading and spelling disorders. However, the highest sensitivity and specificity were obtained for the speed in word reading test (Figure 2). This result, even if preliminary, suggested using as a possible cutoff for speed in reading words a value at CI-FRA total score greater than 75 (having sensitivity = 0.99, and specificity = 0.95).

## Discussion

The main aim of the present study was to propose and test a new screening tool for teachers able to detect early signs of reading and spelling difficulties. To this aim, the dimensionality, validity, sensitivity, and reliability of the CI-FRA checklist were analyzed. The relation between the CI-FRA scores and the results obtained by standardized Italian tests used to assess learning and general cognitive abilities were studied to check for concurrent validity. Preliminary results were obtained with ROC curves to confirm the sensitivity and specificity of the CI-FRA. Findings indicated that CI-FRA shows good internal validity, according to the multifactorial model of reading and spelling disorders. Moreover, high predictive validity, good test–retest reliability, acceptable concurrent validity, and overall adequate psychometric properties were reported.

Exploratory factor analyses provided a three-factor solution representing the dimension of language, phonological awareness, and reading–spelling competences (1), attention (2), and fine motor skills (3), which was confirmed as adequate with CFA. A comparison between the three theoretical models shown as the three-factor model is the one that best represents and fits the original data having all estimated indices close to the expected values. This result suggests that the difficulties emerging in the early stages of reading and spelling acquisition pertain to three different domains. In particular, the three-factor model accomplishes the possible overlapping of specific abilities (phonological domain: reading, spelling, phonological abilities), as well as other important skills investigated (attention and fine motor skills). The model, showing an intermediate specificity along this continuum, could explain the frequent overlapping of different cognitive difficulties in reading and spelling disorders.

The first factor included items related to early reading and spelling abilities (items 4–8 and items 13–15), language and phonological competences (items 1–3), and one item related to short-term verbal ability (item 20). Taken together, these items converge in the dimension of phonological awareness. It is well known that in an early phase, inadequate development of reading, spelling, and language abilities is the principal aspect of the evolution of learning skills. Moreover, verbal memory is strongly linked to linguistic and phonological skills (Adams and Gathercole, 2000) and relates to the difficulty in learning poems, nursery rhymes, and months.

The second factor included items concerning attentional difficulties, mainly related to the tendency to distraction and slight hyperactive attitude (items 16 to 19). Many studies demonstrated the relevant relation existing between attentional problems (in particular visuospatial attention) and learning disorders. Some authors stated that visuospatial attention is decisive in the initial processing of raw visual information that is a process necessary for the elaboration, synthesis, and reading of graphemes, and then a process necessary for the development of reading abilities (Facoetti and Molteni, 2001; Facoetti et al., 2010). Moreover, given the high frequency of visual attention span disorder in dyslexic children, visual attention deficit could be considered as a predictor of future reading difficulties (Valdois et al., 2004; Bosse et al., 2007; Bosse and Valdois, 2009).

The third factor included items related to fine motor skills abilities, specifically regarding difficulties in managing the space in the paper, in fine motricity, and handwriting ability (items 9–12). The predictive role of fine motor skills for the future development of learning abilities (i.e., reading and spelling) has been demonstrated by many studies (Grissmer et al., 2010; Roebers and Jäger, 2014; Cameron et al., 2016) showing that motor skills and language difficulties are often interconnected (Viholainen et al., 2002). Indeed, although motor skills impairment can overlap with executive function deficits (Roebers and Jäger, 2014), an early evaluation of these aspects may still represent a useful indicator of future reading and spelling disorders.

Significant correlations between the CI-FRA scales and scores obtained from standardized tests commonly used to evaluate developmental reading and spelling abilities (measured in the second grade) demonstrated good predictive validity. Moreover, CI-FRA total score and subscale scores correlated with scores obtained from standardized neuropsychological tests. In detail, abilities commonly considered as predictive factors of reading and spelling acquisition (metaphonological and phonological awareness, working memory, phonological memory, speech production) were significantly correlated with all CI-FRA subscales and total score. This confirmed that the CI-FRA scores are in line with those obtained with standardized tests. Even if the correlation analysis results showed moderate effect sizes, these results are in line with former literature investigating the link between different phonological abilities and reading ability (Moll et al., 2014). It is important to mention that, especially in the first 2 years of alphabetization, pupils are highly diverse in the development of reading and spelling abilities. It is therefore not surprising to find high variability in data collected from a primary school sample. Overall, the CI-FRA showed correlations both with domain-specific abilities (i.e., reading and spelling) and with other cognitive abilities (i.e., non-verbal intellectual abilities and motor skills).

Furthermore, even if the sample size was small, the ROC curves showed promising results. Crucially, the CI-FRA total score is highly sensitive for predicting the presence of word reading speed deficit, which is the most important parameter for distinguishing the reading deficit in Italian orthography (Zoccolotti et al., 1999).

## Limitations of the Study

The present preliminary results indicate a promising value of the CI-FRA checklist for primary school teachers. Nevertheless, some limitations should be taken into account. The first limitation relates to the small sample size. The ROC curves are referred to a very small sample of 5 to 10 cases with specific reading or spelling disorder. A larger sample size is therefore required to confirm the sensitivity and specificity here reported. Moreover, a larger clinical sample will allow verifying the consistency of the CI-FRA cutoff score. A second limitation concerns the geographic origin of the sample. All the schools involved in the present study were from the Emilia-Romagna region, located in the north of Italy. Even if the representativeness of the sample compared to the general Italian school population is ensured (see Barbiero et al., 2019), samples from other Italian regions should be included to endure the representativeness of the sample.

## Conclusion

The CI-FRA checklist is conceived as a brief screening tool for teachers for the evaluation of the early signs of reading and spelling disorders. The challenge of a fast tool is to be not only as simple as possible, but also methodologically well-founded. The preliminary evaluation of the psychometric properties of the CI-FRA confirmed that it could be considered a good screening tool for reading and spelling disorders. The CI-FRA includes a general score that could be used as a good indicator of reading and spelling difficulties, as well as specific subscales corresponding to more general abilities (i.e., attention, fine motor, and executive skills) that allow defining the profile of each pupil. The simplicity of the checklist and the reliability allow using the CI-FRA also for the evaluation of the evolution of the pupil’s profile and of the overall class’ composition. The accordance between CI-FRA and cognitive tests highlights the possibility to recognize not only a general fragility in the prerequisites of learning but also the specific early signs for reading and spelling developmental process (Catts et al., 2015).

Importantly, the results of the present research could be considered as preliminary evidence for the development of other checklists for the early screening of learning disorders. Such tools could help teachers to plan early intervention and eventually inform families and clinicians about the possible need for an in-depth evaluation. Crucially, such a tool could represent a significant advantage also for the National Health Service.

## Data Availability Statement

The datasets generated for this study are available on request to the corresponding author.

## Ethics Statement

The research was carried out in accordance with The Code of Ethics of the World Medical Association (Declaration of Helsinki). Written informed consent to participate in this study was provided by the participants’ legal guardian/next of kin.

## Author Contributions

SGi, MB, and LM designed the experiments. SGi, MB, and AA wrote the manuscript. SGi, AA, SM, and MM collected the data. SGi, MB, GC, and SGa performed the data analysis. All authors read and commented on the manuscript.

## Conflict of Interest

The authors declare that the research was conducted in the absence of any commercial or financial relationships that could be construed as a potential conflict of interest.
